# Emphysematous Gastritis in a Patient With Recent COVID-19 Infection

**DOI:** 10.7759/cureus.43270

**Published:** 2023-08-10

**Authors:** Joseph V Vyskocil, James J Vyskocil

**Affiliations:** 1 Critical Care, Central Michigan University College of Medicine, Mount Pleasant, USA; 2 Critical Care, Advocate Aurora St. Luke's Medical Center, Milwaukee, USA

**Keywords:** gastritis, interstitial gastric emphysema, cystic pneumatosis, covid 19, case report, emphysematous gastritis

## Abstract

Emphysematous gastritis, a rare pathology, causes gastric inflammation and intramural gas accumulation due to gas-forming microorganisms. Its diagnosis is made based on high clinical suspicion and confirmed by CT imaging of the stomach, which shows circumferential gas formation along the gastric wall. Early diagnosis and treatment are critical, as emphysematous gastritis is associated with a high mortality rate. Medical treatment consists of bowel rest, hydration, and intravenous broad-spectrum antibiotics. In the event of severe clinical decline despite medical treatment, surgery may be indicated. There may be an association between emphysematous gastritis and coronavirus disease 2019 (COVID-19) infection. We present a case of emphysematous gastritis in a patient with carbapenem-resistant Acinetobacter baumannii (CRAB) and a recent COVID-19 infection.

## Introduction

Emphysematous gastritis is a rare form of gastritis that is caused by gas-forming organisms in the stomach. It is associated with a mortality rate of 55-61% [1,2]. As of January 2023, only 91 cases of this condition have been reported [3]. CT scan is the preferred diagnostic procedure of choice [4], which shows the presence of gas in the wall of the stomach. Emphysematous gastritis is associated with diabetes mellitus, renal failure, recent abdominal surgery, gastroenteritis, nonsteroidal anti-inflammatory drug use, alcohol abuse, and long-term corticosteroid use [1]. There are no official guidelines available for the management of emphysematous gastritis [2], but early diagnosis, initiation of bowel rest, hydration, and intravenous broad-spectrum antibiotics have been noted to improve outcomes [5]. Surgical intervention is not indicated during acute infection because of risks of anastomotic leak, postoperative fistula, and strictures [2]. Surgery should be reserved for patients with signs of clinical deterioration despite optimal medical management, perforations, and uncontrolled disseminated sepsis [6].

## Case presentation

A 68-year-old female was admitted to the hospital and had a multi-week ICU stay for persistent hypotension and acute respiratory failure requiring mechanical ventilator support. On examination, multiple bilateral hemorrhagic bullous wounds were seen on her upper and lower extremities. Blood cultures were positive for Staphylococcus hominis, thought to be secondary to a dialysis catheter infection. During her hospitalization, the patient developed recurrent collapse of her left lung. Skin biopsies of the patient’s bullous wounds revealed carbapenem-resistant Acinetobacter baumannii (CRAB) with New Delhi metallo-beta lactamase by PCR testing. Bronchoscopy washings grew Acinetobacter baumannii as well. The patient completed treatment for her dialysis catheter infection with a 10-day course of vancomycin and was treated with minocycline for suspected Acinetobacter baumannii-associated cellulitis and pneumonia. The patient’s other chronic medical problems included chronic renal failure on hemodialysis, chronic obstructive pulmonary disease, hypertension, diabetes mellitus type 2, and hypothyroidism. The patient also had a history of prolonged prior hospitalization with coronavirus disease 2019 (COVID-19) infection, which had required percutaneous endoscopic gastrostomy (PEG) tube placement and tracheostomy tube placement. The patient had been discharged from her prior prolonged hospitalization six weeks before her present admission. At the time of her readmission to the hospital, both her PEG tube and tracheostomy tube sites were well healed, with no inflammatory changes or signs of infection.

**Figure 1:**
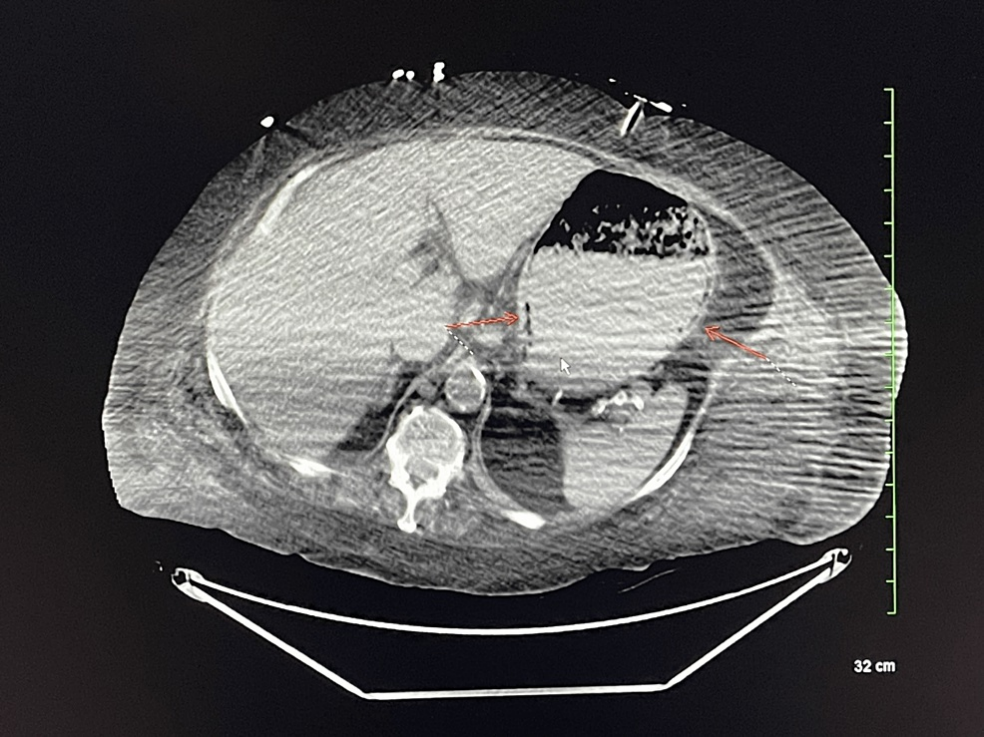
CT of the abdomen slice showing circumferential gas formation (arrows) along the gastric wall CT: computed tomography

After completing antibiotic treatment and while awaiting long-term assisted care, the patient developed clinical worsening with hemodynamic instability requiring vasopressor treatment. The patient also developed leukocytosis (white blood count of 16.0 K/mcL) and lactic acidosis (lactic acid level of 5.4 mmol/L). Aerobic and anaerobic blood cultures were sent. Repeat COVID-19 testing was negative. Fungal cultures were not performed. CT scanning of the chest, abdomen, and pelvis was performed, to look for a new source of infection. CT scan of the abdomen revealed gastric wall circumferential gas formation, diagnostic of emphysematous gastritis (Figure 1). The CT images also revealed no PEG tube tract inflammation or air along the PEG tube tract. Nasogastric tube gastric decompression was performed, tube feedings were placed on hold, and the patient was started on empiric broad-spectrum IV antibiotics with Flagyl, minocycline, and siderophore cephalosporin (cefiderocol). Following treatment, the patient’s lactic acid level and white blood counts decreased, and her hemodynamic instability requiring vasopressor treatment resolved. Cultures drawn prior to initiating antibiotic treatment were non-diagnostic.

## Discussion

Emphysematous gastritis is a rare condition associated with a high mortality rate. If left untreated, it can lead to gastric necrosis, resulting in perforation, sepsis, and death. Morbidity from emphysematous gastritis has not been extensively reported, but the prevalence of gastric strictures secondary to emphysematous gastritis is as high as 25% [3]. Poor prognostic signs include elevated creatinine, elevated serum lactic acid, and pneumatosis involving the small intestine and colon [7]. Organisms associated with emphysematous gastritis include Streptococcus species, Escherichia coli, Enterobacter species, Clostridium species, Pseudomonas aeruginosa, Staphylococcus aureus, Candida species, and Mucor species [5].

The finding of gas in the stomach wall can be seen in interstitial gastric emphysema, cystic pneumatosis, and emphysematous gastritis [8]. Interstitial gastric emphysema is secondary to the introduction of air into the intramural space in the stomach by mechanical shear forces and is self-limited and benign. Cystic pneumatosis reveals gas-filled cysts in the stomach lining, and patients often are either asymptomatic or only mildly symptomatic and have a benign clinical course [9,10]. Hence, patients who appear toxic should be treated with the presumptive diagnosis of emphysematous gastritis when gas is seen in the stomach wall on CT scanning of the abdomen.

There have been only two other documented cases of emphysematous gastritis in patients with COVID-19 infection, apart from one other case in a patient who had recently recovered from COVID-19 infection [3,11,12]. Although the association between emphysematous gastritis and COVID-19 infection remains unclear, the severe acute respiratory syndrome coronavirus 2 (SARS-CoV-2) can take advantage of the gastrointestinal angiotensin-converting enzyme 2 receptors, inducing wall inflammation, barrier compromise, and dysbiosis [13]. Clinical evidence of this effect can be seen in a substantial number of patients with COVID-19 infection who have GI manifestations, sometimes before classic pulmonary symptoms [14]. SARS-CoV-2 may also contribute to the development of emphysematous gastritis by weakening the integrity of the gastric mucosa by causing vascular endothelial cell injury [15].

Our patient had recently recovered from COVID-19 infection, making this the fourth case of emphysematous gastritis in a patient with COVID-19 infection or recent COVID-19 infection. A patient with emphysematous gastritis may present one to six weeks after the triggering event [16]. Hence, it is possible that previous COVID-19 infection contributed to the development of emphysematous gastritis. Although our patient had several risk factors for developing emphysematous gastritis (diabetes mellitus, chronic renal failure, and PEG tube placement), the other reported case of emphysematous gastritis in a patient with recent COVID-19 infection had no specific underlying cause identified [3].

## Conclusions

Emphysematous gastritis is a rare but life-threatening condition. The finding of intramural air on CT of the abdomen in a septic patient helps with the early recognition of the disease and is the procedure of choice for diagnosis. Treatment with bowel rest, hydration, and broad-spectrum intravenous antibiotics with coverage against gram-negative and anaerobic bacteria is required in these patients. Surgical interventions during acute infection are not indicated because of risks of anastomotic leak, postoperative fistula, and strictures. There may be an association between COVID-19 infection or recent infection and the risk of developing emphysematous gastritis.

## References

[1] Takano Y, Yamamura E, Gomi K (2015). Successful conservative treatment of emphysematous gastritis. Intern Med.

[2] Watson A, Bul V, Staudacher J, Carroll R, Yazici C (2017). The predictors of mortality and secular changes in management strategies in emphysematous gastritis. Clin Res Hepatol Gastroenterol.

[3] Ghasemi F, Kirkpatrick I, Sharma A (2023). Medical management of emphysematous gastritis. AIM Clin Cases.

[4] Al-Jundi W, Shebl A (2008). Emphysematous gastritis: case report and literature review. Int J Surg.

[5] Riaz S, Kudaravalli P, Saleem SA, Sapkota B (2020). Emphysematous gastritis: a real indication for emergent surgical intervention?. Cureus.

[6] Sharma P, Akl EG (2016). A combination of intramural stomach and portal venous air: conservative treatment. J Community Hosp Intern Med Perspect.

[7] Jehangir A, Rettew A, Shaikh B, Bennett K, Qureshi A, Jehangir Q (2015). A case report of emphysematous gastritis in a diabetic patient: favorable outcome with conservative measures. J Community Hosp Intern Med Perspect.

[8] Kyawzaw L, Emanuel O, Sandar L, Andrew O, Naing LA, Moshe F, Madhavi R (2018). Case series: gastric emphysema and emphysematous gastritis with air in portal venous system. Gastroenterol Hepatol Open Access.

[9] Johnson PT, Horton KM, Edil BH, Fishman EK, Scott WW (2011). Gastric pneumatosis: the role of CT in diagnosis and patient management. Emerg Radiol.

[10] Misro A, Sheth H (2014). Diagnostic dilemma of gastric intramural air. Ann R Coll Surg Engl.

[11] Garrosa-Muñoz S, López-Sánchez J, González-Fernández LM, Muñoz-Bellvís L (2021). Could SARS-CoV-2 be associated with emphysematous gastritis?. Dig Liver Dis.

[12] Martin IT, Laura CLA, Juan RMJ, Priscilla RMV, Gerardo ZIJ, Antonio ITA (2021). Emphysematous gastritis secondary to gastric mucormycosis in a COVID-19 positive patient: case report. Front Med Case Rep.

[13] Sharma RK, Stevens BR, Obukhov AG (2020). ACE2 (angiotensin-converting enzyme 2) in cardiopulmonary diseases: ramifications for the control of SARS-CoV-2. Hypertension.

[14] Tian Y, Rong L, Nian W, He Y (2020). Review article: gastrointestinal features in COVID-19 and the possibility of faecal transmission. Aliment Pharmacol Ther.

[15] Lowenstein CJ, Solomon SD (2020). Severe COVID-19 is a microvascular disease. Circulation.

[16] Moosvi AR, Saravolatz LD, Wong DH, Simms SM (1990). Emphysematous gastritis: case report and review. Rev Infect Dis.

